# Metformin inhibits pathological retinal neovascularization but promotes retinal fibrosis in experimental neovascular age-related macular degeneration

**DOI:** 10.3389/fphar.2025.1547492

**Published:** 2025-03-20

**Authors:** Xin Wang, Xu Liang, Shiya Huang, Mingyan Wei, Yuan Xu, Xiaodong Chen, Yanliang Miao, Rongrong Zong, Xiang Lin, Shiying Li, Zuguo Liu, Qian Chen

**Affiliations:** ^1^ Xiamen University affiliated Xiamen Eye Center, Fujian Provincial Key Laboratory of Ophthalmology and Visual Science, Fujian Engineering and Research Center of Eye Regenerative Medicine, Eye Institute of Xiamen University, School of Medicine, Xiamen University, Xiamen, Fujian, China; ^2^ Department of Ophthalmology, Suining Central Hospital, Suining, Sichuan, China; ^3^ Department of Ophthalmology, Xiang’an Hospital of Xiamen University, Xiamen, Fujian, China; ^4^ Department of Ophthalmology, The First Affiliated Hospital of Xiamen University, Xiamen, Fujian, China

**Keywords:** metformin, retinal neovascularization, age-related macular degeneration, neovascular AMD, very low-density lipoprotein receptor, retinal fibrosis

## Abstract

**Purpose:**

This study aims to investigate the effects and mechanism of action of metformin on retinal neovascularization and fibrosis in a mouse model of neovascular age-related macular degeneration (nAMD).

**Methods:**

Very low-density lipoprotein receptor knockout (*Vldlr* ^−/−^) mice, a mouse model of nAMD, were used in this study. *Vldlr* ^*−/−*^ mice were administered metformin on postnatal day (P) 20 for 20 days (early stage of pathological change) or at 5.5 months of age for 45 days (late stage of pathological change). Retinal leakage was examined by fundus fluorescein angiography (FFA). Retinal neovascularization was assessed by lectin staining. Retinal fibrosis was assessed by Western blotting, immunofluorescence staining, and Masson’s trichrome staining.

**Results:**

Retinal vascular leakage and neovascularization were significantly reduced in *Vldlr* ^*−/−*^ mice treated with metformin compared to those treated with the vehicle at P40. The protein levels of inflammatory factors and phospho(p)-STAT3 were decreased, and P38 and ERK signaling were suppressed in the retinas of metformin-treated *Vldlr* ^*−/−*^ mice relative to those in the control group at P40. Fibrotic markers were upregulated in the retinas of *Vldlr* ^−/−^ mice treated with metformin compared to those treated with the vehicle at 7 months. Levels of the inflammatory factors and p-STAT3 were increased, and PI3K/AKT, P38, and ERK signaling were upregulated in the retinas of metformin-treated *Vldlr* ^*−/−*^ mice compared to those in the control group at 7 months.

**Conclusion:**

Metformin inhibits pathological retinal neovascularization but promotes fibrosis in experimental nAMD. These results provide evidence and highlight important considerations for the clinical use of metformin in different stages of nAMD.

## Introduction

Age-related macular degeneration (AMD) is the leading cause of blindness in people over 60 (Fleckenstein et al., 2024). Retinal angiogenesis is a pathological change associated with advanced AMD, also known as wet AMD or neovascular AMD (nAMD) (Pugazhendhi et al., 2021). The newly formed vessels damage the highly organized retinal structures, especially the retinal pigment epithelium (RPE) and photoreceptor layers, disrupt retinal function, and cause vision loss (Pugazhendhi et al., 2021). The mechanism of retinal neovascularization is not fully understood. The current treatment strategy relies on anti-vascular endothelial growth factor (VEGF) therapy (Fleckenstein et al., 2024). However, anti-VEGF treatment has several limitations, such as high cost, risk of infection, the need for repeated injections, and diminished efficacy (Wolf et al., 2022). Some patients exhibit incomplete response to anti-VEGF treatment (Mettu et al., 2021). Finding alternative treatments for pathological retinal angiogenesis is an urgent clinical need.

Retinal fibrosis is a pathological change that occurs in the late stages of nAMD (Tenbrock et al., 2022). It usually follows several or multiple episodes of pathological retinal angiogenesis and vascular leakage in patients with nAMD (Tenbrock et al., 2022). Extracellular matrix deposition in the lesions disrupts the normal retinal structure, leading to permanent structural damage and, ultimately, loss of function (Tenbrock et al., 2022). Unfortunately, the mechanism of retinal fibrosis is still unknown, and there is no specific treatment for it (Tenbrock et al., 2022; Armendariz and Chakravarthy, 2024).

Metformin is a clinical drug used to manage blood glucose levels in patients with type 2 diabetes (Bailey, 2017). In addition to its anti-hyperglycemic effects, recent studies have identified its protective effects in many aspects, such as anti-cancer (Vancura et al., 2018), anti-aging (Chen et al., 2022), anti-oxidative stress (Buczyńska et al., 2024), cardioprotective (Bu et al., 2022), and nephroprotective effects (Pan et al., 2020). In addition, multiple preclinical studies have shown that metformin may have therapeutic effects on retinal diseases, such as retinitis pigmentosa (Luodan et al., 2019; Athanasiou et al., 2017), diabetic retinopathy (Kim et al., 2017; Nahar et al., 2021; Yi et al., 2016), ischemic retinopathy (Joe et al., 2015), uveitis (Kalariya et al., 2012), and AMD (Qu et al., 2020; Xu et al., 2018; Ying et al., 2017). However, its effects on retinal neovascularization are not always consistent. For instance, a study showed that metformin suppressed angiogenesis by inhibiting cell proliferation, migration, and tube formation in human retinal vascular endothelial cells (Han et al., 2018). Metformin was reported to inhibit angiogenesis in a laser-induced choroidal neovascularization model (Zhang et al., 2023). However, in a mouse model of oxygen-induced retinopathy (OIR), metformin treatment didn’t reduce the extent of avascular areas at the postnatal day (P)17, and the OIR pathology was remained at P21 even when the vehicle treatment showed significant improvement in OIR pathology at P21 (Joe et al., 2015). These differing effects of metformin on retinal neovascularization in different animal models suggest the complexity of its role in various pathological scenarios.

Recently, several studies have shown that metformin has anti-fibrotic effects. A study reported that metformin reversed well-established lung fibrosis in an adenosine 5′-monophosphate-activated protein kinase (AMPK)-dependent manner in a bleomycin-induced mouse model (Rangarajan et al., 2018). Another study also found that metformin reduced liver collagen deposition, inhibited liver cell apoptosis, and lowered serum malondialdehyde (MDA) levels in a CCl4-induced liver fibrosis model, indicating that metformin exerts anti-fibrotic effects in the liver (Kong et al., 2024). In addition, metformin has been shown to inhibit transforming growth factor-beta (TGF-β) and its downstream signaling, thus reducing TGF-β-induced fibrotic changes (Xiao et al., 2016; Lu et al., 2015; Lim et al., 2012). However, whether metformin has therapeutic effects on retinal fibrosis is still unknown.

In this study, we aim to investigate the effects of metformin on retinal neovascularization and fibrosis in a well-known nAMD model, the very low-density lipoprotein receptor (VLDLR) knockout (*Vldlr* ^−/−^) mice. Metformin was administered to *Vldlr* ^−/−^ mice at two-time points—the early stage of pathological change (the angiogenic stage) and the late stage of pathological change (the fibrotic stage) and the effects of metformin on retinal angiogenesis, vascular leakage, and retinal fibrosis were investigated. Our results indicate that metformin has an anti-angiogenic effect but promotes retinal fibrosis in *Vldlr* ^−/−^ mice. These dual effects of metformin may be mediated through the modulation of multiple signaling pathways, which may play opposing roles in retinal angiogenesis and fibrosis.

## Materials and methods

### Animals

B6; 129S7-Vldlrtm1Her/J (*Vldlr* ^−/−^) mice were obtained from the Jackson Laboratory (Bar Harbor, ME). Wild-type (WT) C57BL/6 J mice were obtained from the Laboratory Animal Center of Xiamen University (Xiamen, China). Age-matched WT mice with similar genetic backgrounds were generated by crossing C57BL/6 J and *Vldlr* ^−/−^ mice. All mice were housed in the Laboratory Animal Center of Xiamen University (Xiamen, Fujian, China). The animal experiments were performed in accordance with the ARVO Statement for the Use of Animals in Ophthalmic and Vision Research, and all studies were conducted in accordance with protocols (XMULAC20220168) approved by the Experimental Animal Ethics Committee of Xiamen University.

### *In vivo* experimental procedure

In the early angiogenic stage, *Vldlr* ^−/−^ mice were treated with metformin (200 mg/kg/day, APExBIO, United States, B1970) or a vehicle solution by daily gavage starting from P20 to P40. After the treatment, the mice underwent fundus fluorescein angiography and electroretinography (ERG), and their eyecups were collected for further experiments at P40. In the late fibrotic stage, a special animal diet containing metformin (at 1333 parts per million) and a control diet were purchased from Medicine Biomedicine Co. Ltd. (Jiangsu, Zhejiang, China). In the late fibrotic stage, *Vldlr* ^−/−^ mice were fed either the metformin-containing diet or vehicle chow starting at 5.5 months of age. After 45 days of feeding, mice were euthanized at 7 months of age, and their eyecups were collected for further experiments.

### Electroretinogram

An ERG system (RetiMINER System; AiErXi Medical Equipment Co., Ltd., Chongqing, China) was used to evaluate the visual function in mice. Mice were dark-adapted overnight and anesthetized with sodium pentobarbital (40 mg/kg). The pupils were dilated with tropicamide phenylephrine eye drops (Santen Pharmaceutical Co., Ltd, Shiga plant, Japan). Full-field ERGs were recorded after subcutaneously inserting a ground electrode near the tail and a reference electrode on the back, followed by placing a golden-ring electrode on the cornea. All procedures were performed under dim red light. The a-wave and b-wave responses to flash stimuli (1.0 cd·s/m^2^) were recorded and analyzed in both eyes. The amplitudes of the oscillatory potential waves were also recorded and analyzed.

### Fundus fluorescein angiography

Mice were anesthetized via intraperitoneal injection of 2% tribromoethanol, and their pupils were dilated with topical 0.5% tropicamide and 0.5% phenylephrine. A 10% fluorescein sodium (Zhiyuan, Tianjin, China) was administered via intraperitoneal injection. The ocular fundus was imaged using a fundus camera (Optoprobe Science, Glamorgan, UK; OPTO-RIS). FFA images were captured 5 min after fluorescein sodium injection.

### Lectin staining

Lectin staining of retinas was performed according to a published protocol (Connor et al., 2009). Briefly, eyeballs were fixed in 4% paraformaldehyde (PFA) for 1 h (h). Retinas were dissected and then incubated with 0.5% Triton X-100 (Sigma-Aldrich) at 4°C overnight. After three washes with 1X PBS, the retinas were incubated with Isolectin GS-IB4 (Thermo Fisher Scientific) overnight at room temperature. Then, the retinas were washed and flat-mounted for microscopy. Quantification of neovascularization was conducted as described previously (Connor et al., 2009).

### Immunofluorescent staining

Eyecups were fixed with 4% PFA and embedded in optimal cutting temperature compound. Frozen sections of 10 μm thickness were fixed in cold acetone (−20°C) for 10 min. Sections were incubated with 0.2% Triton X-100 for 20 min and blocked with 2% BSA in PBS for 1 h. Then, sections were incubated with different primary antibodies at 4°C for 16 h. After three washes with 1X PBS, sections were incubated with Alexa Fluor 594-conjugated IgG (Abcam) or Alexa Fluor 488-conjugated IgG (Abcam) for 60 min at 37°C. Nuclei were counterstained with 4′, 6-diamidino-2-phenylindole (DAPI, Abcam). Images were acquired using a confocal laser scanning microscope (Zeiss, Braunschweig, Germany; LSM 880).

### Hematoxylin and eosin (HE) staining

Fixed eyeballs were dehydrated in ethanol, waxed, and embedded in paraffin. Sections of 6 μm thickness were cut around the optic nerve, followed by deparaffinization in xylene, and rehydration in ethanol. HE staining was performed using a staining kit (Servicebio; G1005) according to the manufacturer’s instructions. The slides were observed under an optical microscope (Zeiss; Axio Lab.A1).

### Western blot analysis

Total protein from eyecups was extracted using RIPA buffer supplemented with protease and phosphatase inhibitors. Protein extracts were separated by 6%–15% SDS–PAGE electrophoresis and transferred onto a polyvinylidene difluoride membrane. The membrane was blocked with 5% non-fat milk for 2 h at room temperature (RT) and incubated with primary antibodies overnight at 4°C. After several washes, the membrane was incubated with a second ary antibody for 1 h at RT. Signal detection was performed using an enhanced chemiluminescence reagent kit (NCM Biotech, Newport, RI, United States). Bands were quantified using ImageJ and normalized to β-actin levels. The following antibodies were used: 5′-adenosine monophosphate (AM)-activated protein kinase (AMPK), phospho(p)-AMPK, p-P38, p-extracellular signal-regulated kinase (ERK), Class I phosphoinositide 3-kinase (PI3K), p-PI3K, protein kinase B (AKT), p-AKT, p-signal transducer and activator of transcription 3 (STAT3), vimentin, collagen-1, connective tissue growth factor (CTGF), glial fibrillary acidic protein (GFAP), and β-actin, which were purchased from Cell Signaling Technology (Danvers, MA, United States). Antibodies such as VEGF and vascular cell adhesion molecule (VCAM-1) were obtained from Santa Cruz Biotechnology (Dallas, TX, United States).

### Statistical analysis

Prism 6 software (GraphPad, San Diego, CA, United States) was used for statistical analysis. A paired Student’s t-test was used for two-group comparisons. Statistical data were expressed as mean ± SEM. p < 0.05 was considered statistically significant.

## Results

### Metformin reduces neovascularization in the retinas of *Vldlr* ^−/−^ mice at P40

**FIGURE 1:**
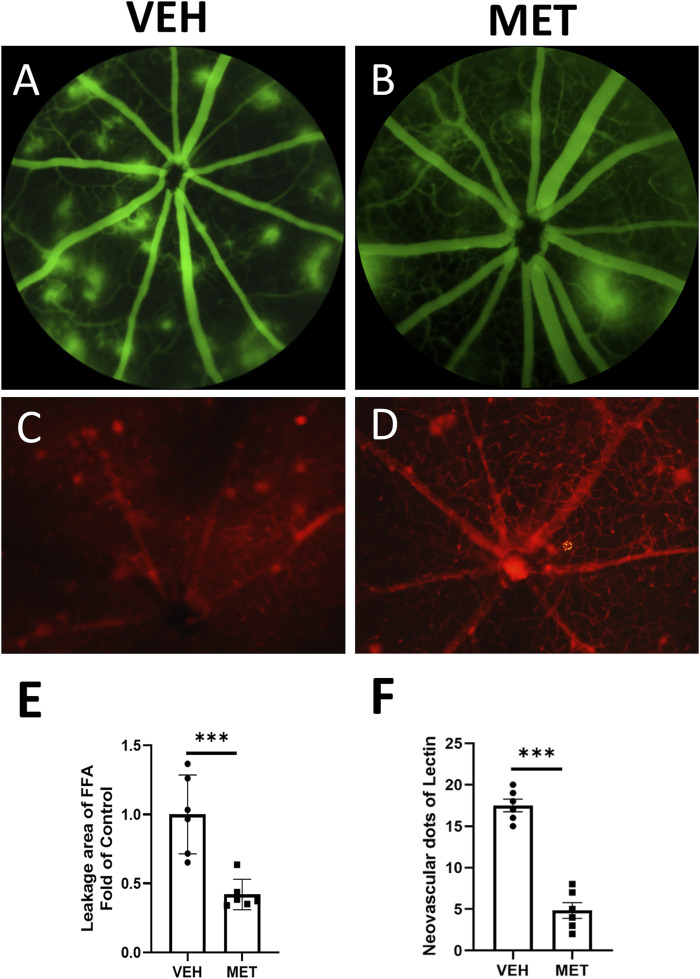
Metformin inhibits retinal vascular leakage and neovascularization in the retinas of *Vldlr* ^−/−^ mice at P40. *Vldlr* ^−/−^ mice were treated with metformin (200 mg/kg/day) or vehicle solution (control) by daily gavage from P20 to P40. The total number of retinal neovascular sprouts was quantified at P40. **(A, B)** Representative images of fundus fluorescein angiography (FFA) of *Vldlr* ^−/−^ mice treated with vehicle (VEH) **(A)** or metformin (MET) **(B)**. **(C, D)** Representative images of lectin staining from *Vldlr* ^−/−^ mice treated with vehicle (VEH) or metformin (MET). **(E, F)** Quantification of leakage areas of FFA images **(E)** or neovascular spots of lectin staining images **(F)** from vehicle and metformin-treated *Vldlr* ^−/−^ mice. Data are shown as mean ± SEM. N = 6, ***p < 0.001. A two-tailed Student’s *t*-test was used.

To assess the effects of metformin on retinal neovascularization, we used *Vldlr* ^−/−^ mice, a mouse model of nAMD (Joyal et al., 2016). Metformin was administered daily by oral gavage from P20 to P40, which corresponds to the early stage of pathological changes dominated by angiogenesis. Vascular leakage was evaluated by FAA (Figures 1A, B). *Vldlr* ^−/−^ mice treated with metformin displayed smaller areas of retinal vascular leakage than those treated with the vehicle solution (Figure 1E). Meanwhile, lectin staining of flat-mounted retinas was performed to detect retinal neovascularization (Figures 1C, D). Metformin treatment significantly reduced the areas of retinal neovascularization in *Vldlr* ^−/−^ mice (Figure 1F). Taken together, these data suggest that metformin suppresses retinal neovascularization and vascular leakage in *Vldlr* ^−/−^ mice at P40.

### Metformin improves oscillatory potentials in the retinas of *Vldlr* ^−/−^ mice at P40

**FIGURE 2:**
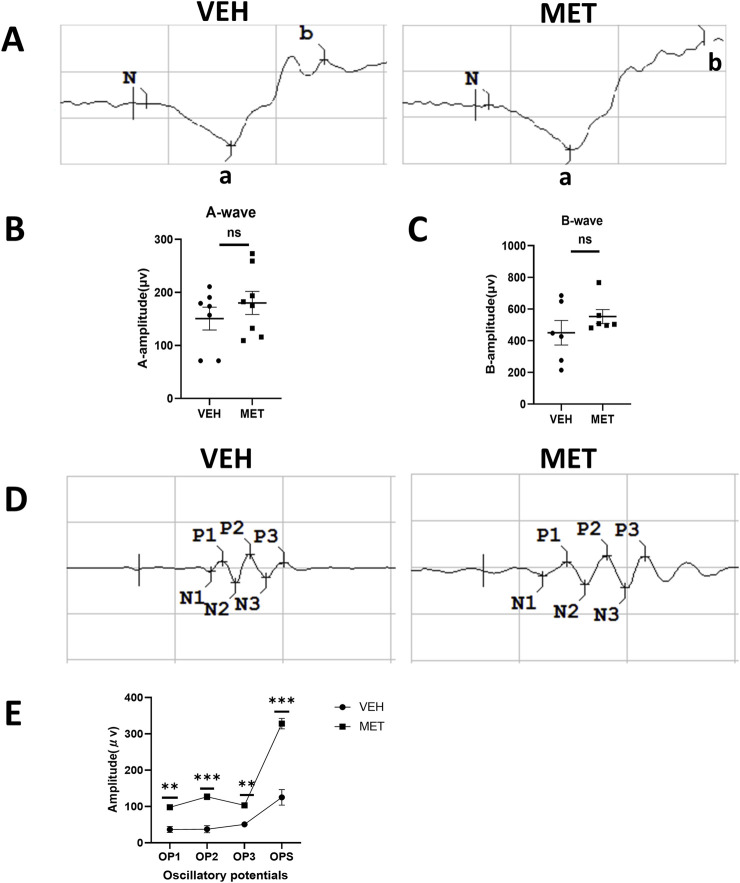
Metformin improves oscillatory potentials in the retinas of *Vldlr* ^−/−^ mice at P40. **(A)** Representative images of the a-wave and b-wave from *Vldlr* ^−/−^ mice treated with vehicle (VEH) or metformin (MET). ERGs were obtained by averaging three responses to 1.0 cd·s/m^2^ flashes. **(B, C)** Amplitudes of ERG a-wave **(B)** and b-wave **(C)** of the two groups were analyzed and quantified. **(D)** Representative images of oscillatory potentials from *Vldlr* ^−/−^ mice treated with vehicle (VEH) or metformin (MET). **(E)** Oscillatory potentials from the two groups were analyzed and quantified. Data are shown as mean ± SEM; n = 6–8, *p < 0.05, **p < 0.01, and ***p < 0.001. A two-tailed Student’s *t*-test was used.

Next, we evaluated whether metformin could improve neuronal function in *vldlr* ^−/−^ mice. Similarly, ERGs, which included the oscillatory potentials, were performed at P40 on *Vldlr* ^−/−^ mice treated with vehicle and *Vldlr* ^−/−^ mice treated with metformin. The a-wave and b-wave showed an upward trend but were not significantly changed after metformin treatment (Figures 2A–C). However, the oscillatory potentials were partially rescued by metformin treatment (Figures 2D, E). Overall, metformin improves the oscillatory potentials in the retinas of *Vldlr* ^−/−^ mice, suggesting a potential improvement in the blood supply to the inner retina.

### Metformin reduces pro-inflammatory factors and inhibits P38 and ERK signaling in the eyecups of *Vldlr* ^−/−^ mice at P40

**FIGURE 3:**
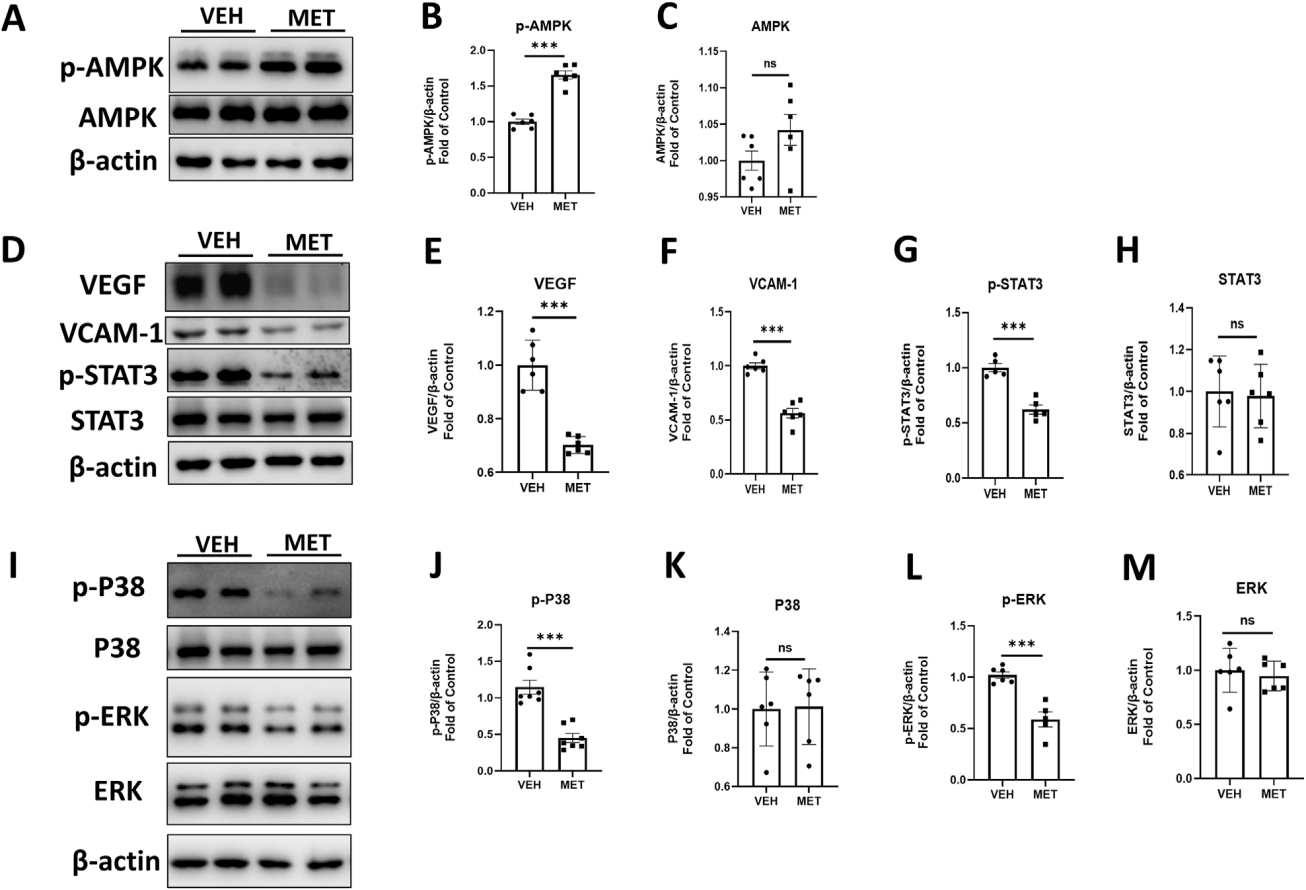
Metformin reduces the retinal pro-inflammatory cytokines, p-P38 and p-ERK, in an AMPK-dependent manner in the eyecups of *Vldlr* ^−/−^ mice at P40. **(A–C)** The protein levels of p-AMPK **(A, B)** and AMPK **(A, C)** in the eyecups of *Vldlr* ^−/−^ mice treated with vehicle (VEH) or metformin (MET) were determined by Western blot analysis and quantified by densitometry. **(D–H)** The protein levels of VEGF **(D, E)**, VCAM-1 **(D, F)**, p-STAT3 **(D, G),** and STAT3 **(D, H)** in the eyecups of *Vldlr* ^−/−^ mice treated with vehicle (VEH) or metformin (MET) were determined by Western blot analysis and quantified by densitometry. **(I–M)** Protein levels of p-P38 **(I, J)**, P38 **(I, K)**, p-ERK **(I, L),** and ERK **(I, M)** in the eyecups of the two indicated groups were determined by Western blot analysis and quantified by densitometry. Data are shown as mean ± SEM; n = 6. *p < 0.05, **p < 0.01, and ***p < 0.001. A two-tailed Student’s *t*-test was used.

Multiple studies have shown that metformin acts as an AMPK agonist, exerting its effects through the activation of AMPK signaling (Zhou et al., 2001; He and Wondisford, 2015). However, many studies suggest that metformin may play its role independent of AMPK signaling (Foretz et al., 2010; Bridges et al., 2014). To determine whether metformin activates AMPK in this study, we assessed the protein levels of p-AMPK and AMPK by Western blot analysis (Figure 3A). The levels of p-AMPK were significantly increased after metformin administration (Figure 3B), while the levels of total AMPK remained unchanged (Figure 3C). These findings suggest a possible mechanism by which metformin activates AMPK signaling in the retina. Furthermore, we investigated the specific mechanism of action of metformin in the eyecups of *Vldlr* ^−/−^ mice. The protein levels of VEGF, VACM-1, and p-STAT3 were significantly decreased in the retina of metformin-treated *Vldlr* ^−/−^ mice (Figures 3D–G), while the levels of total STAT3 were not significantly changed (Figures 3D, H). This suggests that metformin may inhibit retinal inflammation and reduce p-STAT3 in *Vldlr* ^−/−^ mice. In addition, protein levels of p-P38 and p-ERK were reduced in *Vldlr* ^−/−^ retinas (Figures 3I, J, L), while the levels of P38 and ERK were unchanged (Figures 3I, K, M).These findings suggest that the P38 and ERK pathways may play a role in the metformin-mediated effects in the eyecups of *Vldlr* ^−/−^ mice.

### Metformin promotes subretinal fibrosis in *Vldlr* ^−/−^ mice at 7 months of age

**FIGURE 4:**
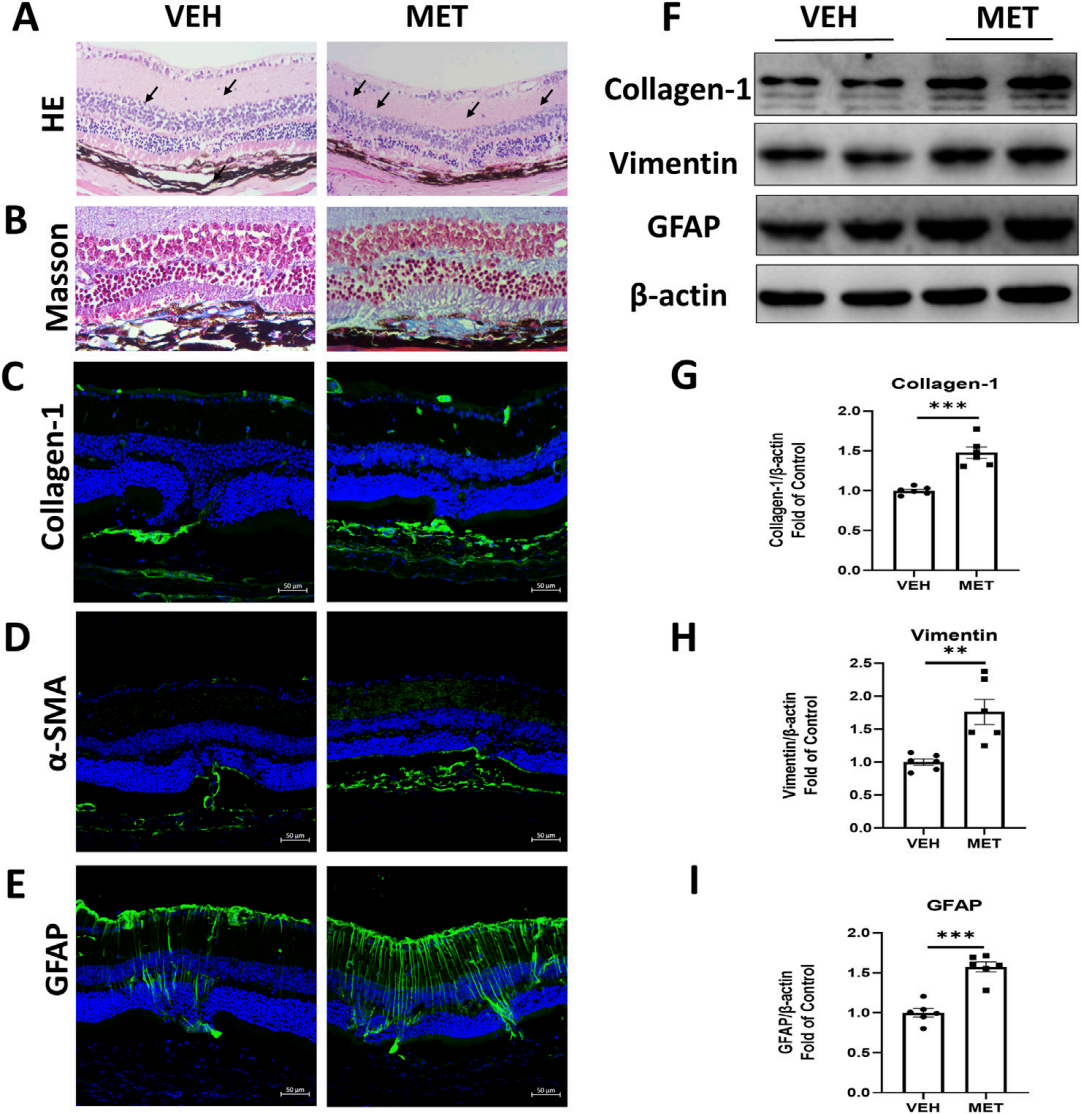
Metformin promotes subretinal fibrosis in *Vldlr* ^−/−^ mice at 7 months of age. *Vldlr* ^−/−^ mice were fed with a diet containing metformin from the age of 5.5 months to 7 months. The mice were euthanized at 7 months of age. **(A, B)** Representative retinal images of H&E staining **(A)** and Masson’s staining **(B)** of collagen deposition in the retinal paraffin sections of *Vldlr* ^−/−^ mice fed with control chow (VEH) or metformin chow (MET). **(C–E)** Representative images of immunostaining show the expression of collagen-1 **(C)**, α-SMA **(D)**, and GFAP **(E)** in the retinal cryosections of the two indicated groups. **(F–I)** The protein levels of collagen-1**(F, G)**, vimentin **(F, H)**, and GFAP **(F, I)** were determined by Western blot analysis and quantified by densitometry in the two indicated groups. Data are shown as mean ± SEM; n = 6. *p < 0.05 and **p < 0.01. A two-tailed Student’s *t*-test was used.

To further explore the function of metformin, we tested whether metformin could suppress retinal fibrosis in the eyecups of *Vldlr* ^−/−^ mice. The *Vldlr* ^−/−^ mice at the age of 5.5 months were treated with metformin for 45 days, and the expression levels of fibrotic markers were assessed. HE staining of retinal sections showed that *Vldlr* ^−/−^ mice treated with metformin have more lesion sites than those treated with the vehicle (Figure 4A). Masson’s staining showed that more blue-stained collagens were deposited in the sub-retinal area of *Vldlr* ^−/−^ mice treated with metformin (Figure 4B). In addition, immunostaining showed that the signals of collagen-1 (Figure 4C), α-SMA (Figure 4D), and GFAP (Figure 4E) were stronger in *Vldlr* ^−/−^ mice treated with metformin than those in the control group. The protein levels of fibrotic markers, collagen-1 and vimentin, were significantly upregulated in the eyecups of metformin-treated *Vldlr* ^−/−^ mice (Figures 4F–H). Expression of GFAP, a glial activation marker, was elevated in the eyecups of *Vldlr* ^−/−^ mice treated with metformin (Figures 4F, I). Taken together, these data suggest that metformin promotes subretinal fibrosis in *Vldlr* ^−/−^ mice.

### Metformin increases inflammation in the eyecups of *Vldlr* ^−/−^ mice at 7 months of age

**FIGURE 5:**
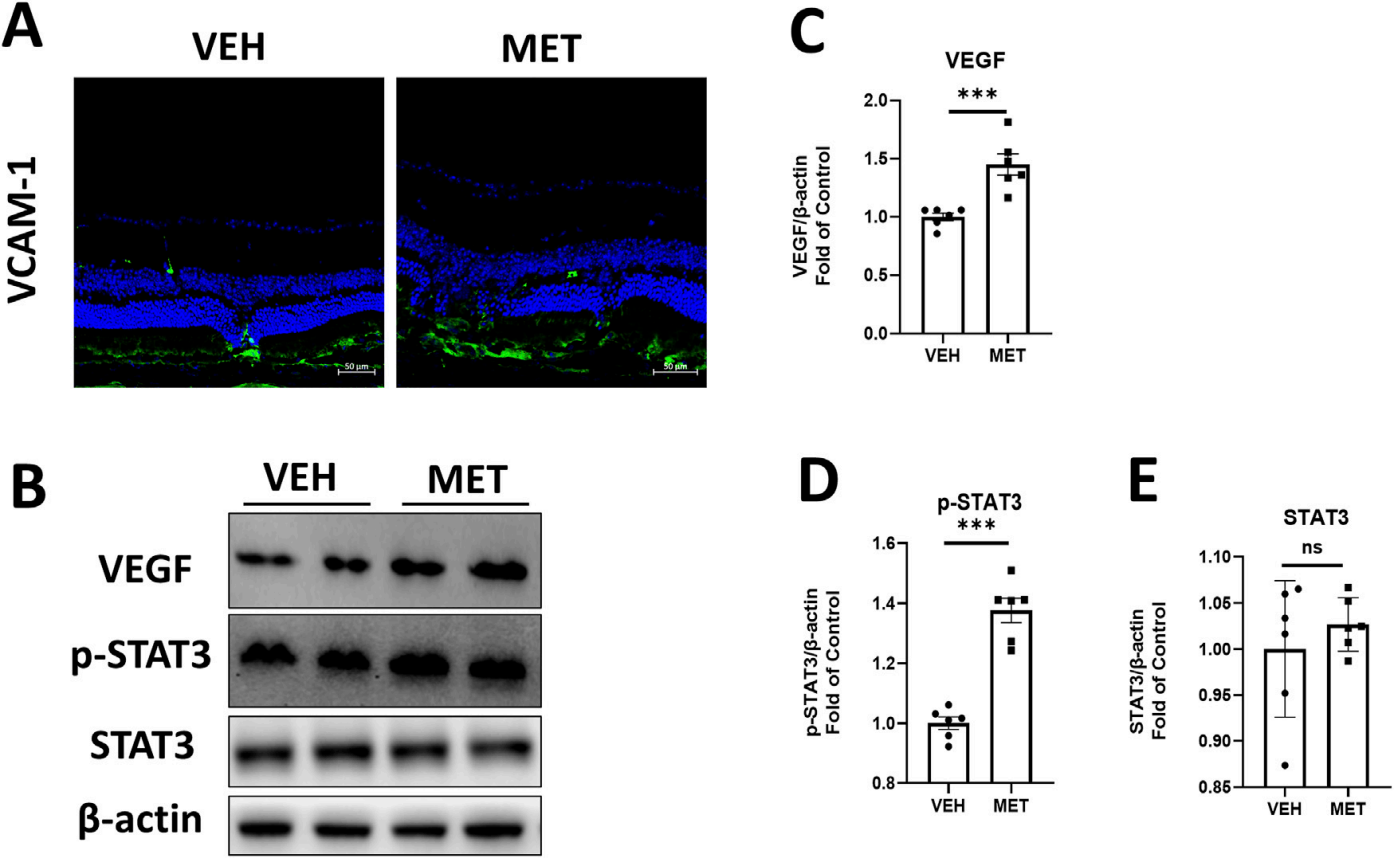
Metformin increases inflammation in the eyecups of *Vldlr* ^−/−^ mice at 7 months of age. **(A)** Representative images of immunostaining show the expression of VCAM-1 in the cryosection of *Vldlr* ^−/−^ mice fed with vehicle (VEH) or metformin (MET). **(B–E)** The protein levels of VEGF **(B, C)**, p-STAT3 **(B, D)**, and STAT3 **(B, E)** in the *Vldlr* ^−/−^ mice fed with control chow (VEH) or metformin chow (MET) were determined by Western blot analysis and quantified by densitometry. Data are shown as mean ± SEM; n = 6. *p < 0.05 and **p < 0.01. A two-tailed Student’s *t*-test was used.

Next, we explored the possible mechanism by which metformin promotes retinal fibrosis in *Vldlr* ^−/−^ mice. Immunostaining showed an increased signal for VCAM-1 in the retinal cryosections of *Vldlr* ^−/−^ mice treated with metformin (Figure 5A). Furthermore, the protein levels of VEGF (Figures 5B, C) and p-STAT3 (Figures 5B, D) were elevated in the eyecups of metformin-treated *Vldlr* ^−/^ mice, while the total protein levels of STAT3 remained unchanged (Figures 5B, E). These findings suggest that metformin may increase retinal inflammation in *Vldlr* ^−/−^ mice at 7 months of age.

### Metformin activates PI3K/AKT, P38, and ERK signaling in the eyecups of *Vldlr* ^−/−^ mice at 7 months of age

**FIGURE 6:**
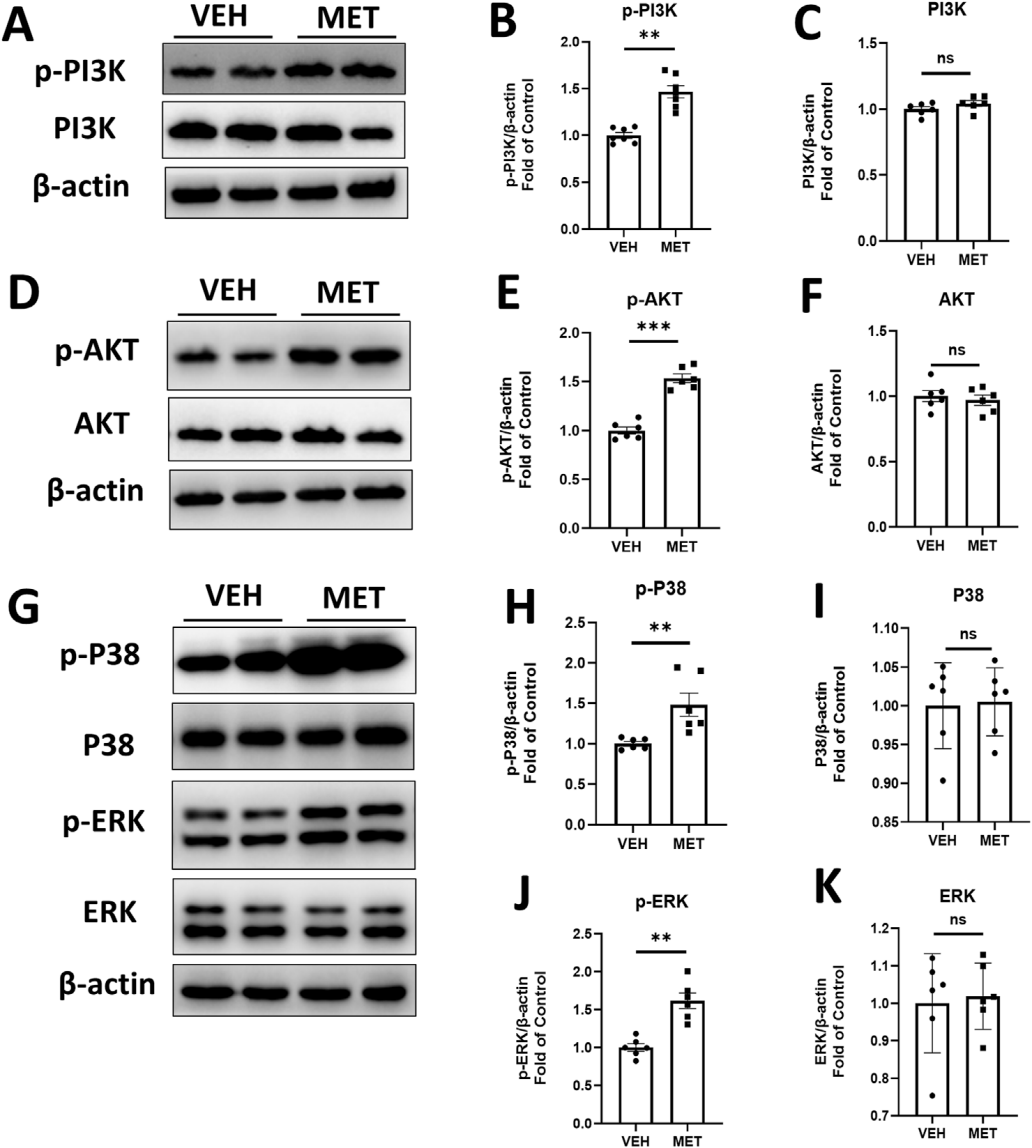
Metformin activates PI3K/AKT, p-P38, and p-ERK pathways in the eyecups of *Vldlr* ^−/−^ mice at 7 months of age. (A–C) The protein levels of p-PI3K **(A, B)** and PI3K **(A, C)** in the *Vldlr* ^−/−^ mice fed with control chow (VEH) or metformin chow (MET) were determined by Western blot analysis and quantified by densitometry. **(D–F)** The protein levels of p-AKT **(D, E)** and AKT **(D, F)** in the two indicated groups were determined by Western blot analysis and quantified by densitometry. **(G–K)** The protein levels of p-P38 **(G, H)**, P38 **(G, I)**, p-ERK **(G, J)**, and ERK **(G, K)** in the two indicated groups were determined by Western blot analysis and quantified by densitometry. Data are shown as mean ± SEM; n = 6. *p < 0.05 and **p < 0.01. A two-tailed Student’s *t*-test was used.

Furthermore, we examined several signaling pathways in the eyecups of *Vldlr* ^−/−^ mice treated with vehicle or metformin. Interestingly, the protein levels of p-PI3K were significantly increased, while the total PI3K levels remained unchanged in the eyecups of *Vldlr* ^−/−^ mice treated with metformin (Figures 6A–C). Similarly, the protein levels of p-AKT were increased, whereas the total AKT levels remained unchanged (Figures 6D–F). In addition, the protein levels of p-P38 (Figures 6G, H) and p-ERK (Figures 6G, J) were elevated in the eyecups of *Vldlr* ^−/−^ mice treated with metformin, with no changes observed in the protein levels of P38 and ERK (Figures 6G, I, K). Taken together, these results suggest that metformin activates the PI3K/AKT, P38, and ERK signaling pathways in the eyecups of *Vldlr* ^−/−^ mice.

## Discussion

Metformin has been reported to have therapeutic effects in non-diabetic diseases such as cancer, cardiovascular disease, and lung fibrosis (Foretz et al., 2023). In this study, we investigated the effects of metformin on retinal neovascularization and retinal fibrosis in *Vldlr* ^−/−^ mice, a model of nAMD (Joyal et al., 2016; Chen et al., 2020). In the early pathological stage, metformin inhibited retinal vascular leakage and neovascularization in *Vldlr* ^−/−^ mice by suppressing inflammatory factors and modulating P38 and ERK signaling pathways. In contrast, during the late pathological stage, metformin promoted retinal fibrosis in *Vldlr* ^−/−-/-^ mice by enhancing inflammation and activating the PI3K/AKT, P38, and ERK signaling pathways. These differential effects of metformin on retinal angiogenesis and fibrosis highlight the complex role of metformin in retinal diseases, providing valuable insights and considerations for its clinical use at different stages of AMD.

According to the pathological changes, AMD can be divided into two types: dry AMD and wet AMD (Fleckenstein et al., 2024; Boopathiraj et al., 2024). Wet AMD, also known as neovascular AMD (nAMD), is an advanced stage of AMD characterized by retinal neovascularization (Pugazhendhi et al., 2021; Boopathiraj et al., 2024). The *Vldlr* ^*−/−*^ mouse model is considered an animal model for retinal angiomatous proliferation (RAP), a special type of nAMD (Hu et al., 2008). In our previous study, we observed elevated fibrotic markers such as collagen-1, vimentin, and fibronectin, along with collagen deposits in the eyes of 6-month-old *Vldlr* ^−/−^ mice (Chen et al., 2020). These findings suggest that *Vldlr* ^−/−^ mice could be used as a mouse model for studying retinal fibrosis. Moreover, *Vldlr* ^−/−^ mice have also been used as a model for retinal fibrosis in many other reports (Yang et al., 2024; Ma et al., 2024).

Based on our long-term investigation and other reports, we have artificially divided the two stages according to the pathological vascular changes in *Vldlr* ^−/−^ mice in this study. The first stage is from P20 to P40, and the second stage spans from 5.5 months to 7 months. The P20–P40 period in *Vldlr* ^−/−^ mice is considered an early stage of pathological change, dominated by angiogenesis. In contrast, the 5.5–7-month period is regarded as the late stage of pathological changes, characterized by predominant fibrosis. These two time periods were specially chosen to mimic the different stages of nAMD observed in clinical settings.

In the early stage of the pathological process, metformin could inhibit retinal vascular leakage and neovascularization in *Vldlr* ^*−/−*^ mice, indicating its inhibitory effects on retinal angiogenesis. These results were consistent with those of several studies that reported the ability of metformin to suppress retinal neovascularization (Han et al., 2018; Zhang et al., 2023). For instance, a study showed that metformin suppressed angiogenesis by inhibiting cell proliferation, migration, and tube formation in human retinal vascular endothelial cells while also reducing inflammatory molecules induced by tumor necrosis factor α (Han et al., 2018). Zhang et al. (2023) showed that oral metformin inhibited laser-induced choroidal neovascularization and decreased macrophage/microglia infiltration. Metformin has also been found to reduce the stability of hypoxia-inducible factor-1α (HIF-1α), decreasing its accumulation under hypoxic conditions and lowering VEGF expression (Wang et al., 2015).

Metformin has been implicated in patients with AMD. For instance, metformin use in AMD patients without diabetes has been explored (Aggarwal et al., 2024; Brown et al., 2019). In a retrospective case-control study, Emily et al. reported that patients who had taken metformin showed decreased odds of developing AMD (Brown et al., 2019). Similarly, another study found that exposure to metformin was associated with reduced odds of developing AMD, and its use was also associated with decreased odds of developing dry AMD (Aggarwal et al., 2024). However, the majority of the reported retrospective case-control studies did not distinguish between dry and wet AMD. Therefore, it is very challenging to assess the effects of metformin on wet AMD in the literature. Using an animal model of nAMD, our study showed that metformin may benefit patients in the early stage of wet AMD when retinal angiogenesis is predominant. More studies, especially prospective clinical trials, are needed to further investigate the protective role of metformin in wet AMD.

More importantly, our study demonstrated the effects of metformin on retinal fibrosis. Surprisingly, we found that metformin promoted retinal fibrosis in *Vldlr* ^−/−^ mice at 7 months of age. These findings are in contrast with other studies that have shown the protective effects of metformin against fibrosis in other diseases. For instance, Kheirollahi et al. (2019) reported that metformin exerts potent protective effects against lung fibrosis by inhibiting TGF-β-mediated fibrosis, suppressing collagen production, and modulating lipogenic differentiation. In addition, studies have shown that metformin has anti-fibrotic effects in the kidney, liver, and heart (Wu et al., 2021). Since our study was based on *Vldlr* ^−/−^ mice, further studies are warranted to include additional animal models of retinal fibrosis to better validate the effects of metformin.

The mechanisms of metformin on retinal angiogenesis and retinal fibrosis were investigated in this study. In the angiogenic stage, metformin inhibited VEGF and p-STAT3 and suppressed P38 and ERK signaling. The inhibitory effects of metformin on STAT3, P38, and ERK signaling pathways have been supported by many studies (Deng et al., 2012; Xia et al., 2021; Rice et al., 2009; Zhao et al., 2022). For example, Deng et al. (2012) reported that metformin inhibits STAT3 activation (p-STAT3) and downstream signaling in parental cell lines. Metformin has been reported to promote NK cell activity in a p38 MAPK-dependent manner (Xia et al., 2021). Studies have also shown that metformin could regulate the ERK-mediated pathway (Rice et al., 2009; Zhao et al., 2022). However, in our study, we found that metformin promoted VEGF and p-STAT3 and increased PI3K/AKT, P38, and ERK signaling in the fibrotic stage. The opposite effects on inflammation and multiple signaling pathways in two different stages of nAMD may indicate the complexity of the role of metformin in treating eye diseases. The possible mechanism is that metformin may help maintain homeostasis by balancing the upregulated and downregulated signaling pathways throughout the pathological process of nAMD. This assumption is supported by many other studies showing that metformin may have opposing effects. For example, studies indicate that metformin promotes endothelial cell proliferation and increases the density of new blood vessels in the spinal cord (Zhao et al., 2023). It has also been found to enhance the angiogenic capacity and autophagy of human adipose tissue-derived stem cells (Tao et al., 2023). In contrast, other studies have shown that metformin inhibits VEGF and angiogenesis. For example, a study showed that metformin inhibited tumor angiogenesis and reduced VEGF expression in implanted murine breast cancer models (Wang et al., 2018). The paradoxical effects of metformin on angiogenesis suggest that its actions may vary in an organ-dependent or disease-dependent manner.

In summary, our study demonstrates that metformin inhibits retinal neovascularization in the early stage and promotes retinal fibrosis in the late stage of nAMD by differentially regulating PI3K/AKT, P38, and P-ERK signaling pathways. These results provide evidence and highlight important considerations for the clinical use of metformin in different stages of nAMD.

## Data Availability

The raw data supporting the conclusions of this article will be made available by authors on request.
